# A bibliometric analysis of immune escape in colorectal cancer: research trends, key contributors, and future directions

**DOI:** 10.3389/fimmu.2025.1614613

**Published:** 2025-06-27

**Authors:** Bowen Zha, Jiahui He, Chunguang Li

**Affiliations:** Department of Gastroenterology, Daxing District Integrated Traditional Chinese and Western Medicine Hospital, Beijing, China

**Keywords:** immune escape, colorectal cancer, bibliometric analysis, immunotherapy, tumor microenvironment

## Abstract

**Background:**

Colorectal cancer (CRC) has brought a serious disease burden to the whole world. Immune escape not only promotes the growth and metastasis of CRC, but also limits the effect of immunotherapy. The purpose of this study is to clarify the research status of immune escape in CRC through bibliometrics.

**Methods:**

This analysis examined publications on immune escape in CRC from the Web of Science Core Collection. The time limit is 2015-2024. After searching and screening by two researchers, data were collected and various analysis were conducted using tools such as VOSviewer, CiteSpace, and bibliometrix. By analyzing the large-scale existing literature data and using the quantitative method of bibliometric analysis, the research trends and emerging topics can be effectively identified.

**Results:**

A total of 573 articles and reviews were included. From 2015-2024, the annual growth rate of 15.93%. The research from China is the most (50.09%), but the research from the United States and Germany is cited more times. Frontiers in Immunology has published the most articles (6.46%). Lei Wang and Peter J.K. Kuppen have made notable contributions, with substantial international collaboration. Keyword analysis highlights research hotspots such as tumor microenvironment and immune-related signaling pathways.

**Conclusion:**

The latest research status of immune escape in CRC is shown. Understanding the immune escape mechanism is very important for understanding the occurrence and development of CRC and developing effective immunotherapy strategies. Future research directions include integrating multiple databases to reduce biases inherent in single-database analyses and employing machine learning methods to predict emerging research hotspots, thus providing actionable insights into the dynamic landscape of immune escape research in CRC.

## 1 Introduction

Colorectal cancer (CRC) is one of the most common malignant tumors in the world, which brings a serious disease burden to the whole world (1–3). Immune escape plays an important role in CRC, which is the mechanism by which tumor cells escape the detection and destruction of host immune system, thus promoting tumor growth and metastasis (4–7). On the other hand, immune escape will also affect the effectiveness of immunotherapy by affecting immune checkpoints and tumor microenvironment (8–10).

Despite the increasing recognition of immune escape’s critical role, detailed molecular pathways and effective therapeutic strategies in CRC remain elusive. More and more studies have realized the importance of immune escape in the occurrence, development and treatment of CRC (11–14). Therefore, the comprehensive analysis of research trends and the determination of influential research and hot directions are very important to guide future research work. Moreover, there is a lack of comprehensive analysis of the research status and future direction of immune escape in CRC.

The purpose of this study is to make a bibliometric analysis of the research prospect of CRC immune escape, and to check the trend, main contributors and emerging research topics. Through bibliometric and visual analysis of the publications of Web of Science (WOS) from 2015 to 2024, we try to describe the evolution of this field by analyzing the research status in this field, and emphasize the potential direction of future research.

## 2 Method

### 2.1 Database and search strategy

**Figure 1:**
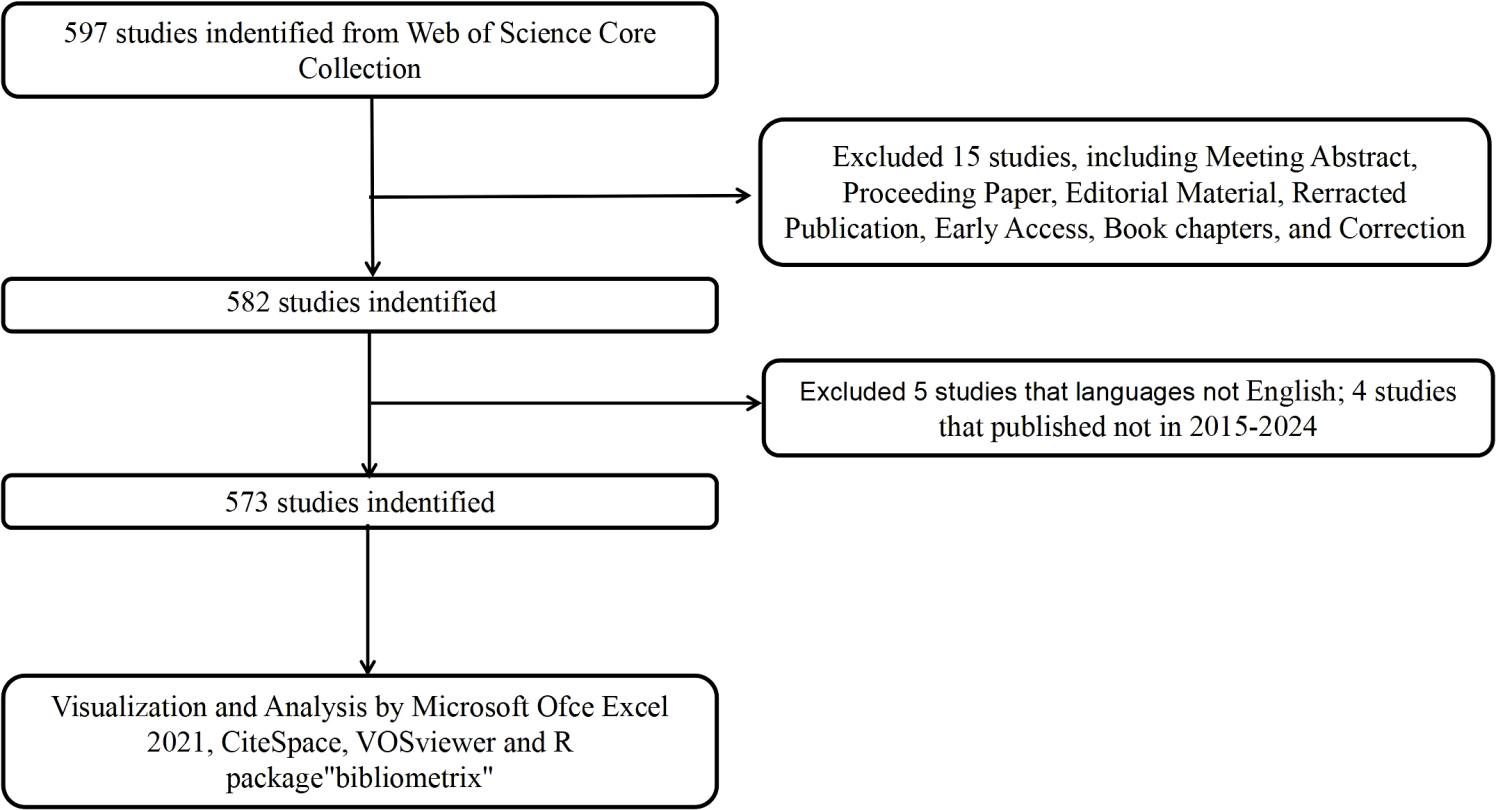
Flowchart of the screening process.

The literature for this bibliometric analysis was retrieved from the Web of Science Core Collection, covering the period from January 1, 2015, to December 31, 2024. To minimize discrepancies caused by ongoing updates to the database, all searches were performed on a single day (February 12, 2025). The initial search yielded 597 records. For the purposes of this study, only articles and reviews published in English were included, while other types of publications such as reprints, book chapters, conference abstracts, and news articles were excluded. After applying these inclusion criteria, 573 documents were retained for the analysis, consisting of 383 articles and 190 reviews. The detailed screening process is shown in **Figure 1**. Boolean operators were utilized to construct the query. The initial broad search was refined through manual screening based on abstracts and titles to include only studies explicitly addressing immune escape mechanisms in CRC, thus ensuring topic specificity. To mitigate bias from incomplete annual data, normalization by monthly averages was considered, and explicit acknowledgment of partial-year inclusion was noted in interpretation. To ensure the accuracy and reliability of the results, two researchers independently conducted the literature search and data collection.

### 2.2 Data collection

We extracted key data from the selected publications, including journal name, publication year, title, authors, country/region, affiliations, abstract, keywords, and references. Journal impact factor (IF) and Journal Citation Reports (JCR) category were obtained from the Web of Science. The productivity metric was based on citation count. After exporting the data, we performed cleaning by merging duplicates and correcting any spelling errors manually. The cleaned data was then analyzed further. To ensure accuracy and reliability, two authors independently extracted the data.

### 2.3 Data analysis

In this study, we utilized specialized tools including the bibliometrix package (https://www.bibliometrix.org) in R software (version 4.4.3), VOSviewer (version 1.6.20), and CiteSpace (version 6.2.R4) to data visualization and analysis (15–17). To ensure data accuracy and reliability, two authors independently conducted the data extraction and analysis, which minimized potential errors and biases, thereby improving the quality and credibility of the study findings.

## 3 Result

### 3.1 An overview of publications

**Figure 2:**
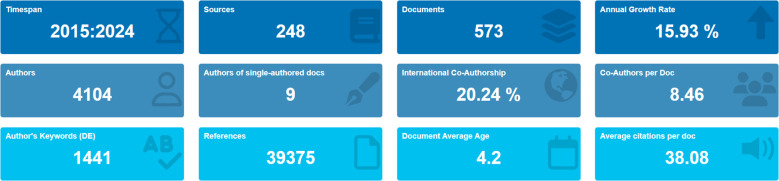
Basic information of research of Immune escape in CRC.

The current status of immune escape in CRC is illustrated in **Figure 2** using R-bibliometrix. The analysis includes publications from 248 journals, with an annual growth rate of 15.93%. A total of 4,104 authors contributed to these publications, with 9 articles authored by a single individual. Articles involving international collaboration accounted for 20.24% of the total. On average, each article had 8.46 authors, 1,441 author keywords, and 39,375 references cited. The average citation lifespan of each paper, from being first noticed to becoming less recognized, was 4.2 years, and each article had been cited an average of 38.08 times.

### 3.2 The annual trend in the number of publications

**Figure 3:**
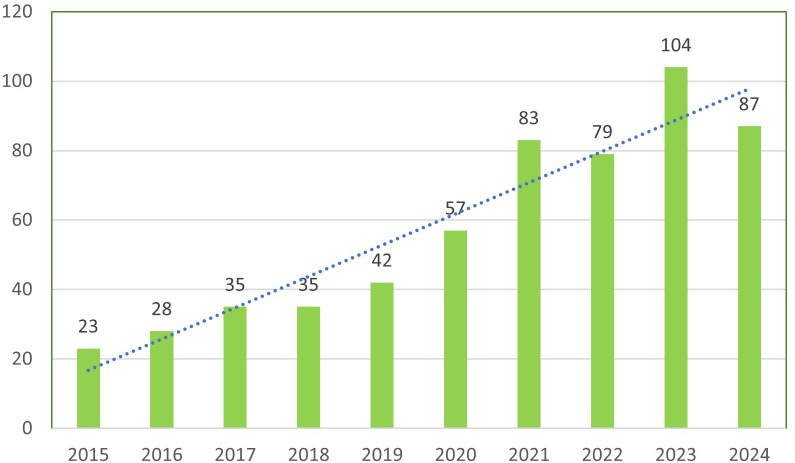
Annual output of research of Immune escape in CRC.

The analysis of annual publications on immune escape in CRC shows a steady increase in research output over the past decade. The number of publications grew from 23 in 2015 to a peak of 104 in 2023, followed by a slight decline to 87 in 2024. This indicates consistent growth in the volume of research, with the highest publication counts observed in 2023 and 2021, as illustrated in **Figure 3**. Regarding citation impact, the mean citations per article exhibited considerable fluctuations. In 2015, articles averaged 64.09 citations, which increased to 92.09 in 2018. However, from 2021 onward, citation counts gradually declined, with a significant drop to 3.51 in 2024. The observed citation decline post-2021 may reflect the natural lag in citation accrual. To validate this observation, future studies could incorporate metrics such as citation half-life or field-normalized citation scores to better contextualize recent citation dynamics. As shown in **Table 1** and **Figure 3**.

**Table 1:** Average Citations Per Year of Immune escape in CRC.

Year	MeanTCperArt	N	MeanTCperYear
2015	64.09	23	5.83
2016	45.50	28	4.55
2017	84.54	35	9.39
2018	92.09	35	11.51
2019	73.38	42	10.48
2020	54.42	57	9.07
2021	33.81	83	6.76
2022	21.37	79	5.34
2023	18.36	104	6.12
2024	3.51	87	1.75

### 3.3 Country and institution analysis

The bibliometric analysis of source countries reveals significant variations in the distribution of publications related to immune escape in CRC. China leads the field with a substantial margin, contributing 287 articles, which accounts for 50.09% of the total publications. The USA follows with 57 articles (9.95%), while Italy (34 articles, 5.93%), Germany (30 articles, 5.24%), and France (20 articles, 3.49%) round out the top five contributors. Other notable countries include Japan (15 articles, 2.62%), the United Kingdom (12 articles, 2.09%), Australia (11 articles, 1.92%), the Netherlands (10 articles, 1.75%), and Canada (9 articles, 1.57%), as presented in **Table 2**.

**Table 2:** Top 10 countries and institutions on research of Immune escape in CRC.

Rank	Country	Counts (%)	Institution	Counts (%)
1	CHINA	287(50.09%)	SUN YAT SEN UNIVERSITY	57(9.95%)
2	USA	57(9.95%)	FUDAN UNIVERSITY	47(8.20%)
3	ITALY	34(5.93%)	UNIVERSITE PARIS CITE	39(6.81%)
4	GERMANY	30(5.24%)	CENTRAL SOUTH UNIVERSITY	38(6.63%)
5	FRANCE	20(3.49%)	INSTITUT NATIONAL DE LA SANTE ET DE LA RECHERCHE MEDICALE	36(6.28%)
6	JAPAN	15(2.62%)	NANJING MEDICAL UNIVERSITY	35(6.11%)
7	UNITED KINGDOM	12(2.09%)	SHANGHAI JIAO TONG UNIVERSITY	35(6.11%)
8	AUSTRALIA	11(1.92%)	HARVARD UNIVERSITY	29(5.06%)
9	NETHERLANDS	10(1.75%)	ASSISTANCE PUBLIQUE HOPITAUX PARIS	27(4.71%)
10	CANADA	9(1.57%)	GERMAN CANCER RESEARCH CENTER	27(4.71%)

In terms of citation impact, China has an average of 28.40 citations per article. Despite having fewer publications, the USA demonstrates a much higher citation average, with 81.40 citations per article. Germany and Italy also show significant citation impact, with Germany averaging 57.10 citations per article and Italy 40.50 citations per article. These figures indicate that while China dominates in publication volume, countries like the USA and Germany have a higher citation impact per article, reflecting the global influence and visibility of their research contributions. The higher citation impact observed in research from the USA and Germany compared to China may be attributed to stronger international collaboration networks, preferences for publication in journals with wider global readership, and higher engagement in open-access publishing, all of which potentially enhance visibility and influence.

The analysis of institutional contributions identifies the leading institutions in this field. Sun Yat-sen University ranks first with 57 publications, comprising 9.95% of the total, followed closely by Fudan University with 47 articles (8.20%). Université Paris Cité (39 articles, 6.81%) and Central South University (38 articles, 6.63%) rank third and fourth, respectively. The Institut National de la Santé et de la Recherche Médicale (36 articles, 6.28%) ranks fifth, followed by Nanjing Medical University and Shanghai Jiao Tong University, each contributing 35 articles (6.11%). Harvard University, with 29 publications (5.06%), ranks eighth, while Assistance Publique - Hôpitaux de Paris and the German Cancer Research Center, each with 27 publications (4.71%), complete the top ten, as shown in **Table 2**.

**Figure 4:**
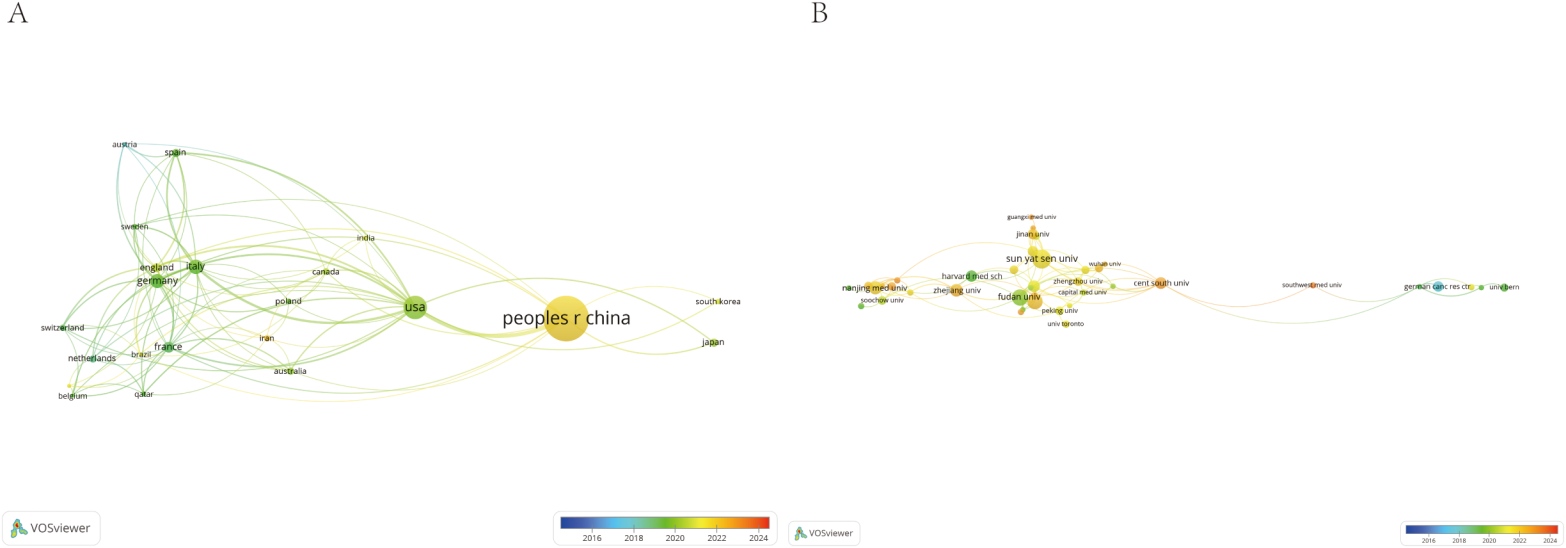
The visualization of country **(A)** and institutions **(B)** on research of Immune escape in CRC, which indicated that these countries and institutions play a crucial role in the research in this field.

Subsequently, we used VOSviewer to analyze the collaboration network among countries and institutions that published more than five studies. This analysis visualized the relationships based on the number and contacts of publications, creating a collaborative network (**Figure 4**). Notably, most countries and institutions demonstrate extensive cooperation. The figure also highlights the major publishing years of different countries and institutions, revealing that certain countries or institutions, such as China and Sun Yat-sen University, are emerging as key players in this research domain.

### 3.4 Journals and co-cited journals analysis

The bibliometric analysis of immune escape in CRC highlights the top ten journals contributing to the literature, with Front Immunol (6.46%), Cancers (5.58%), and Front Oncol (4.01%) being the leading sources. These journals are highly respected in immunology and oncology, all classified in the Q1 quartile based on impact factors. The analysis also identified the most frequently co-cited journals, including Cancer Research (1476 citations, IF 12.5, Q1), Nature (1173 citations, IF 50.5, Q1), and Clinical Cancer Research (1106 citations, IF 10.4, Q1). These journals play a pivotal role in advancing the understanding of immune escape mechanisms in cancer, reflecting the substantial influence of high-impact publications in shaping the current research landscape in this field. As shown in **Table 3**.

**Table 3:** Top 10 journal and Co-cited journal of Immune escape in CRC.

Rank	Journal	Counts (%)	IF (2024) /JCR division	Co-cited journal	Co-citation	IF (2024)/JCR division
1	Front Immunol	37 (6.46%)	5,7 (Q1)	Cancer Res	1476	12.5 (Q1)
2	Cancers	32 (5.58%)	4.5 (Q1)	Nature	1173	50.5 (Q1)
3	Front Oncol	23 (4.01%)	3.5 (Q2)	Clin cancer res	1106	10.4 (Q1)
4	J Immunother Cancer	15 (2.62%)	10.3 (Q1)	Cell	909	45.6 (Q1)
5	Oncoimmunology	14 (2.44%)	6.5 (Q1)	Front Immunol	859	5,7 (Q1)
6	Int J Mol Sci	12 (2.09%)	4.9 (Q1)	Science	853	44.8 (Q1)
7	Cancer Lett	8 (1.40%)	9.1 (Q1)	J Immunol	767	3.6 (Q2)
8	Front Cell Dev Biol	8 (1.40%)	4,6 (Q1)	Proc Natl Acad Sci U S A	744	9.4 (Q1)
9	Int J Cancer	8 (1.40%)	5,7 (Q1)	N Engl J Med	679	96.3 (Q1)
10	Cancer Res	7 (1.22%)	12.5 (Q1)	J Clin Oncol	677	42.1 (Q1)

**Figure 5:**
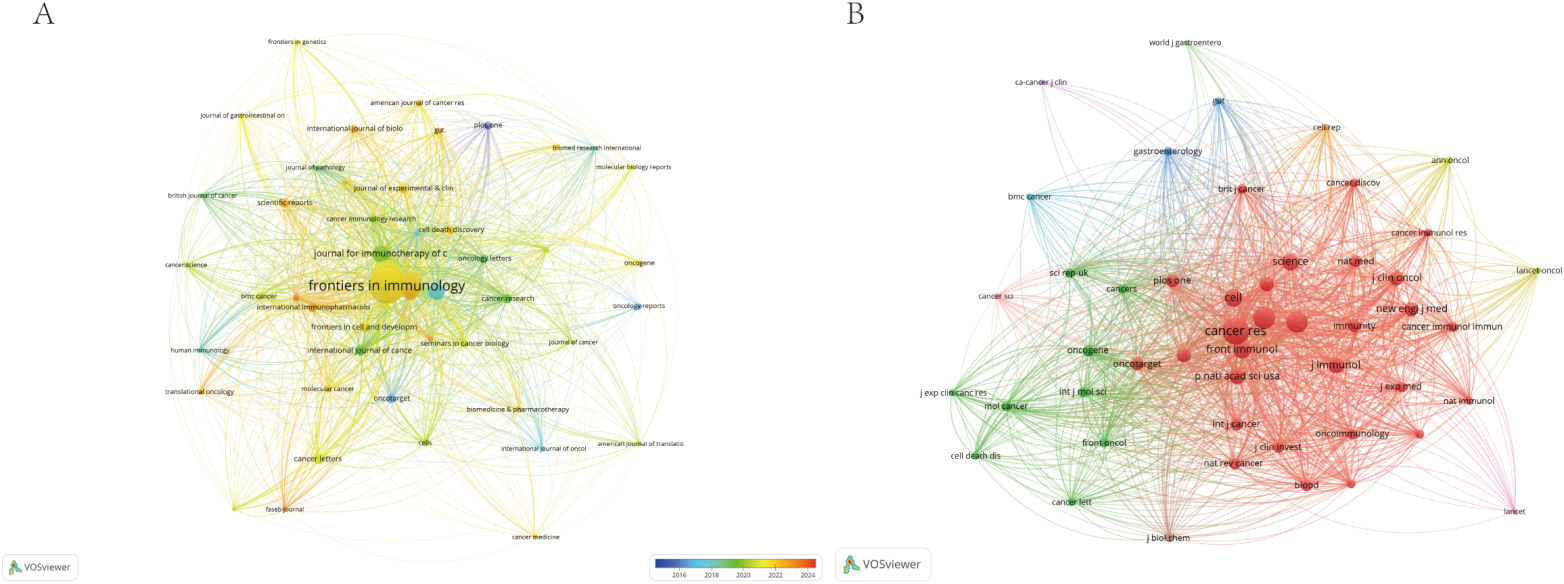
The visualization of journals **(A)** and co-cited journals **(B)** of research of Immune escape in CRC.

Additionally, we used VOSviewer to analyze and visualize the relationships between journals and co-cited journals. The resulting journal network and co-citation network, depicted in **Figure 5**, illustrate the active citation interactions among key periodicals in the research on immune escape in CRC. This network visualization highlights the collaborative nature of the field and underscores the centrality of these prominent journals in the ongoing development of this area of study.

### 3.5 Author and co-cited author analysis

The analysis of authorship in the field of immune escape in CRC identifies the top contributors and their key collaborators. Lei Wang leads the field with 7 articles and 238 citations, closely followed by Peter J.K. Kuppen, who has published 6 articles with 212 citations. Prominent co-cited authors include Le, D.T. (128 co-citations) and Galon, J. (92 co-citations), highlighting their significant influence in the area. Additionally, Pornpimol Charoentong stands out with 4 articles and 720 citations, and Wei Wang has contributed 6 articles with 219 citations. Both researchers are frequently co-cited with renowned experts such as Hanahan, D. (88 co-citations) and Overman, M.J. (76 co-citations). The analysis also emphasizes the critical role of collaborative networks, with influential co-cited authors like Topalian, S.L. (72 co-citations) and Siegel, R.L. (72 co-citations) shaping the research direction. As shown in **Table 4**.

**Table 4:** Top 15 authors and co-cited author of Immune escape in CRC.

Rank	Author	Article	Citation	Article/document	Co-Cited Author	Co-Citation
1	wang, lei	7	238	34	le, dt	128
2	kuppen, peter j. k.	6	212	35.3	galon, j	92
3	wang, wei	6	219	36.5	hanahan, d	88
4	charoentong, pornpimol	4	720	180	overman, mj	76
5	liu, yang	4	440	110	sung, h	74
6	wang, yan	4	104	26	siegel, rl	72
7	li, wei	4	96	24	topalian, sl	72
8	klement, john d.	4	92	23	zhang, y	67
9	liu, kebin	4	92	23	liu, y	65
10	lu, chunwan	4	92	23	mantovani, a	65
11	yang, dafeng	4	92	23	zhang, l	64
12	swets, marloes	4	91	22.8	wang, y	58
13	zhang, xueguang	4	84	21	li, y	56
14	seliger, barbara	4	64	16	gabrilovich, di	51
15	yang, li	4	42	10.5	pagès, f	51

**Figure 6:**
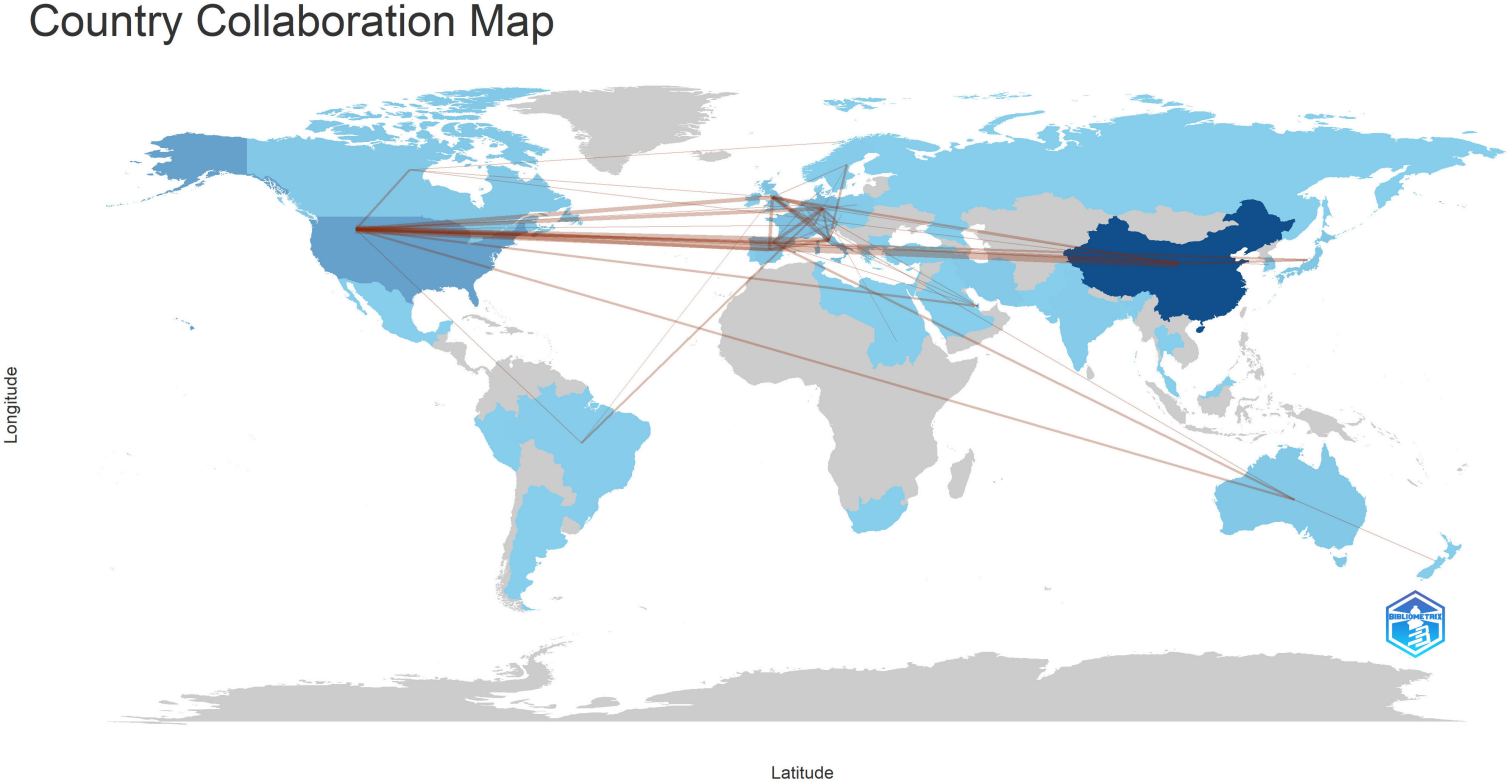
The Geographic distribution of publications and their international collaborations.

**Figure 7:**
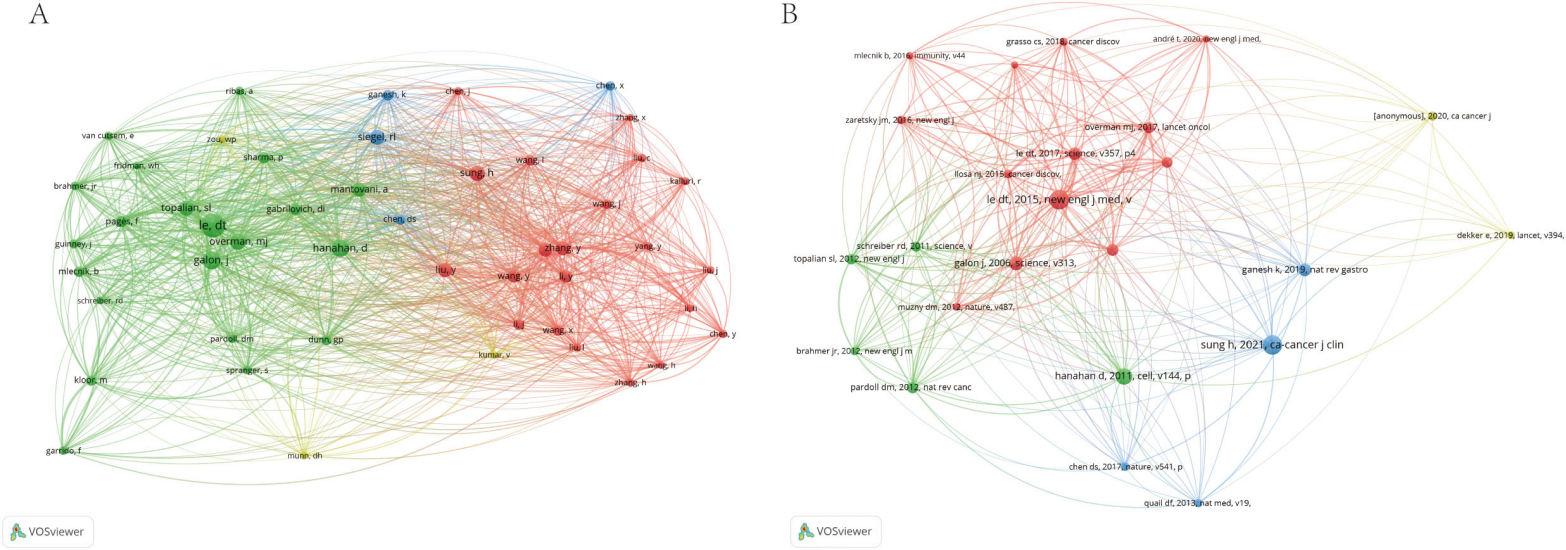
The visualization of co-cited author **(A)** and references **(B)** on research of Immune escape in CRC.

In terms of international collaboration, **Figure 6** illustrates the cooperative networks among countries, underscoring the strong academic ties between researchers in China and North America. This map reveals an established and extensive network of cross-border research collaboration, reflecting the global nature of efforts to advance the understanding of immune escape in CRC. **Figure 7** highlights the interactions between co-cited authors and articles, reinforcing the importance of international cooperation in tackling the complex challenges presented by immune escape mechanisms in cancer research. These findings suggest that such global partnerships are instrumental in driving progress in this critical area of study.

### 3.6 Keyword analysis

**Figure 8:**
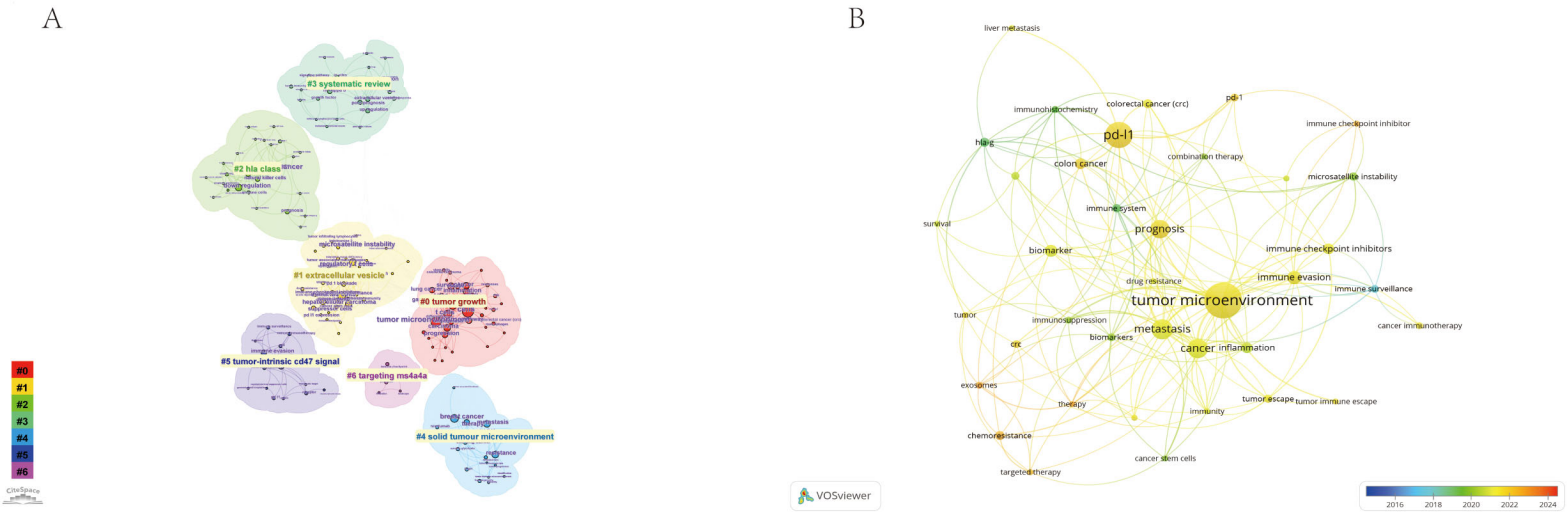
The Keyword cluster analysis  **(A)** and visualization of keywords **(B)** on research of Immune escape in CRC. #0: tumor growth; #1: extracellular vesicles; #2: HLA class; #3: systematic reviews; #4:solid tumor microenvironment; #5:tumor-intrinsic CD47 signaling; #6: targeting MS4A4A.

Using the CiteSpace parameters, a network was generated to visualize the relationships between research topics in the field of immune escape in CRC. The Cluster ID denotes the specific cluster number, with clusters labeled as #0, #1, #2, and so on. The size of each cluster corresponds to the number of publications within it. In **Figure 8A**, seven distinct clusters were identified, each representing a key research theme, including tumor growth, extracellular vesicles, HLA class, systematic reviews, solid tumor microenvironment, tumor-intrinsic CD47 signaling, and targeting MS4A4A. These clusters encapsulate the central topics in the field, offering insights into the diverse areas of focus within immune escape research. Notably, identified keywords such as CD47 signaling and MS4A4A have direct translational implications, linking fundamental research to ongoing clinical trials in immunotherapy. For instance, targeting CD47 is actively being investigated in clinical trials aiming to overcome immune resistance, underscoring the clinical relevance of these research hotspots. Additionally, **Figure 8B** presents the close relationships between keywords, as analyzed using VOSviewer.

### 3.7 Trend analysis

**Figure 9:**
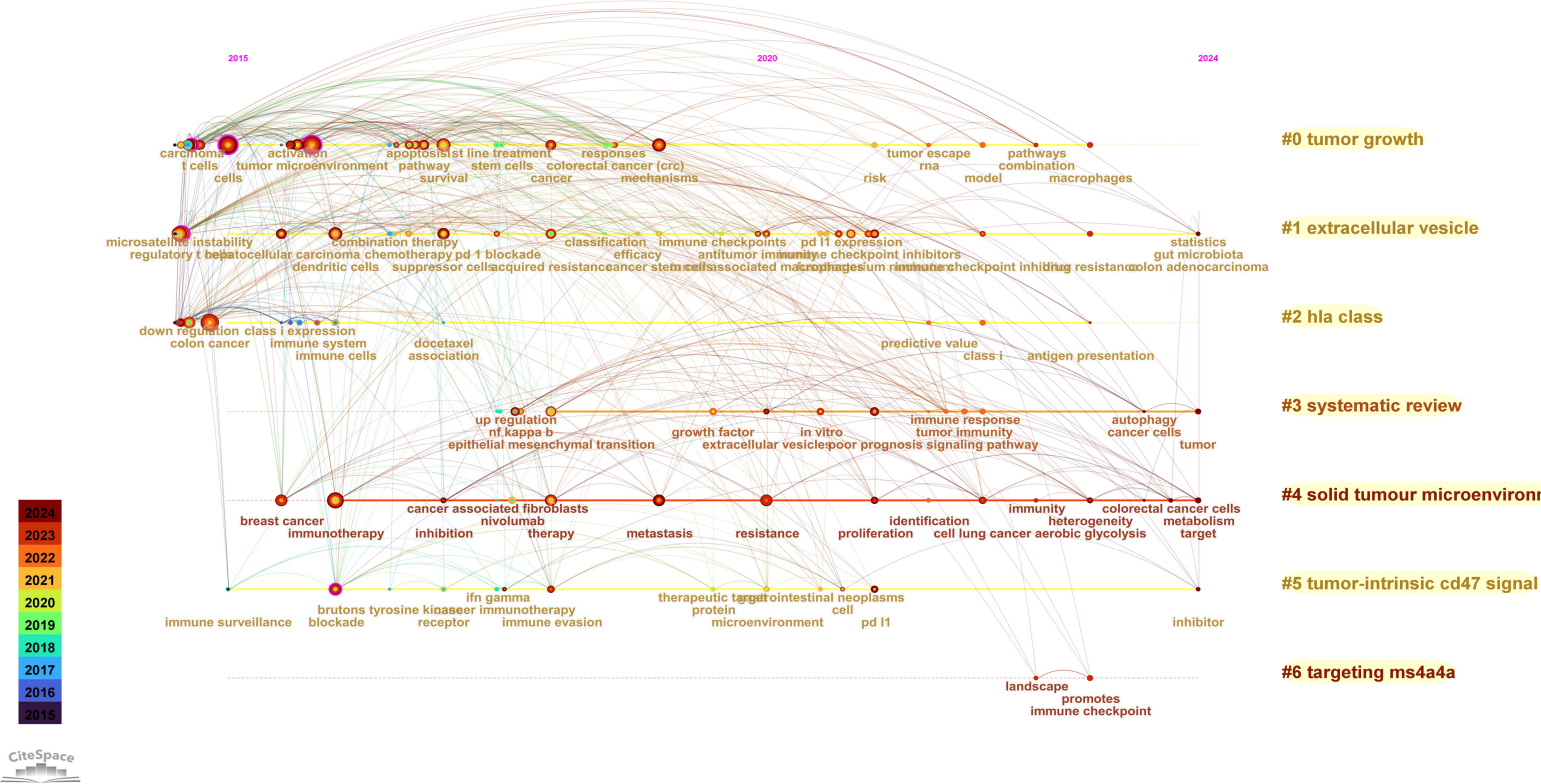
The timeline view of keywords analysis on research of Immune escape in CRC.

**Figure 10:**
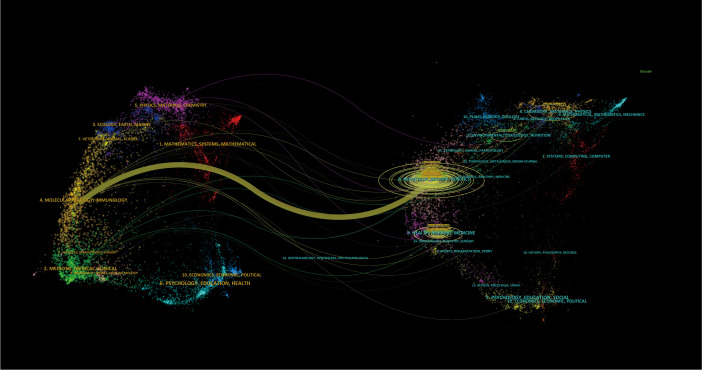
The dual-map overlay of journals of Immune escape in CRC.

The Timeline viewer provides insight into the evolving trends of research hotspots by tracking keywords and analyzing the temporal patterns within clusters, illustrating the development of popular research topics over time. Documents grouped in a cluster are placed on a horizontal line, with time moving from left to right, reflecting the significance and volume of research in each field. **Figure 9** visually depicts the key research areas and future directions for immune escape in CRC. **Figure 10** shows that citing journals primarily come from the fields of Molecular Biology, Immunology, Medicine, and Clinical studies, referred to as research frontiers, while the cited journals are mainly from Molecular Biology and Genetics, forming the knowledge base. The dual-map overlay of journals suggests that the hotspots and frontiers of immune escape in CRC research will increasingly concentrate on Molecular Biology and Immunology.

## 4 Discussion

Immune escape plays a critical role in the progression and metastasis of CRC, significantly influencing the prognosis and therapeutic response of patients. The ability of tumor cells to evade immune surveillance is a major barrier to effective treatment, particularly with immunotherapies (18–21).

Our bibliometric analysis reveals a growing global interest in immune escape mechanisms in CRC, with a notable rise in publications from 2015 to 2024, peaking in 2023. While China dominates in publication volume, countries like the USA and Germany exhibit higher citation rates, indicating their concentrated research influence. Strong academic collaborations, particularly between China and North America, are pivotal in advancing this field. Leading journals such as Front Immunol and Cancers, along with key co-cited journals like Cancer Res and Nature, highlight the integration of immune escape mechanisms into mainstream cancer research. Influential authors like Lei Wang and Peter J.K. Kuppen, alongside prominent co-cited researchers such as D.T. Le and J. Galon, shape the research trajectory, underscoring the importance of international partnerships in cancer immunotherapy.

Keyword analysis and trend observations, particularly through the use of tools like CiteSpace and VOSviewer, reveal key research areas such as tumor microenvironment, extracellular vesicles (EV), and HLA class signaling. These clusters reflect emerging research topics that are likely to define future research directions. The shift in research from broad immunological concepts toward more specific mechanisms, such as CD47 signaling and targeting MS4A4A, illustrates a refinement in the focus of immune escape research in CRC.

Tumor growth is a fundamental process in cancer progression, driven by both genetic mutations within tumor cells and their ability to manipulate the surrounding tumor microenvironment. Tumor cells have been shown to recruit immune suppressive cells and secrete cytokines that promote tumor growth while inhibiting immune responses. The ability of tumor cells to evade immune detection and destruction is critical to sustained tumor progression (22–24). A growing body of evidence suggests that the interaction between tumor cells and the extracellular matrix contributes to tumor growth, survival, and resistance to treatment. Specifically, extracellular vesicles play a crucial role in the communication between tumor cells and the immune system (25). These vesicles can transport various molecules such as proteins, lipids, and RNA, facilitating immune escape by modulating immune cell function and promoting metastasis. Tumor-derived EVs have been implicated in the reprogramming of immune cells, such as dendritic cells and macrophages, to a more immunosuppressive phenotype, further enhancing immune escape (26, 27).

Another critical aspect of immune escape in CRC is the role of HLA class molecules in tumor immune surveillance. The downregulation or loss of HLA class I molecules on tumor cells impairs their recognition by cytotoxic T lymphocytes, one of the primary mechanisms by which tumors evade immune destruction (28–30). Moreover, the solid tumor microenvironment is inherently immunosuppressive, characterized by a high degree of hypoxia, low pH, and the accumulation of immunosuppressive cells such as regulatory T cells and myeloid-derived suppressor cells. These factors collectively contribute to the inhibition of effective immune responses against the tumor (31, 32).

The tumor-intrinsic CD47 signaling pathway is another pivotal mechanism in immune escape. CD47, often referred to as the “don’t eat me” signal, interacts with the signal regulatory protein alpha (SIRPα) on macrophages, preventing phagocytosis (33, 34). This interaction is particularly important in the context of immune escape in solid tumors, where the overexpression of CD47 on tumor cells helps them evade macrophage-mediated clearance. Targeting CD47 or its receptor SIRPα has emerged as a promising therapeutic strategy to enhance anti-tumor immunity (5, 35, 36). Additionally, the growing interest in targeting MS4A4A has highlighted its potential role in modulating the immune response. MS4A4A is a protein associated with immune cell activation, has been shown to be involved in immune escape, and targeting this protein could restore immune function and enhance the efficacy of cancer immunotherapies (37). This presents a novel avenue for therapeutic intervention, particularly in cancers with high immune escape potential like CRC.

There are some limitations that should be considered. First, the scope of the analysis is limited to publications indexed in the Web of Science Core Collection, which may exclude relevant literature available in other databases or non-English language publications. This restriction may introduce a bias towards studies published in high-impact journals and those with global accessibility. Incorporating additional databases like Scopus or PubMed and non-English literature could further enhance comprehensiveness and reduce potential publication bias. Additionally, the analysis focused solely on articles and reviews, excluding other types of publications such as conference proceedings or book chapters, which may contain valuable insights. The reliance on citation-based metrics, such as the number of citations per article, may also skew the results, as newer articles may not have had sufficient time to accrue citations. Therefore, while citation counts offer an important measure of research impact, they may not fully reflect the recent influence of emerging studies.

## Data Availability

The original contributions presented in the study are included in the article/supplementary material. Further inquiries can be directed to the corresponding author.

## References

[1] Huang J Lucero-Prisno DE 3rd Zhang L Xu W Wong SH Ng SC. Updated epidemiology of gastrointestinal cancers in East Asia. Nat Rev Gastroenterol Hepatol. (2023) 20:271–87. doi: 10.1038/s41575-022-00726-3 36631716

[2] Sung H Jiang C Bandi P Minihan A Fidler-Benaoudia M Islami F. Differences in cancer rates among adults born between 1920 and 1990 in the USA: an analysis of population-based cancer registry data. Lancet Public Health. (2024) 9:e583–e93. doi: 10.1016/S2468-2667(24)00156-7 39095135

[3] Wang Y Huang Y Chase RC Li T Ramai D Li S. Global burden of digestive diseases: A systematic analysis of the global burden of diseases study, 1990 to 2019. Gastroenterology. (2023) 165:773–83.e15. doi: 10.1053/j.gastro.2023.05.050 37302558

[4] Al Zein M Boukhdoud M Shammaa H Mouslem H El Ayoubi LM Iratni R. Immunotherapy and immunoevasion of colorectal cancer. Drug Discov Today. (2023) 28:103669. doi: 10.1016/j.drudis.2023.103669 37328052

[5] Luo Q Shen F Zhao S Dong L Wei J Hu H. LINC00460/miR-186-3p/MYC feedback loop facilitates colorectal cancer immune escape by enhancing CD47 and PD-L1 expressions. J Exp Clin Cancer Res. (2024) 43:225. doi: 10.1186/s13046-024-03145-1 39135122 PMC11321182

[6] Mao Y Xu Y Chang J Chang W Lv Y Zheng P. The immune phenotypes and different immune escape mechanisms in colorectal cancer. Front Immunol. (2022) 13:968089. doi: 10.3389/fimmu.2022.968089 36032084 PMC9399611

[7] Stein A Simnica D Schultheiß C Scholz R Tintelnot J Gökkurt E. PD-L1 targeting and subclonal immune escape mediated by PD-L1 mutations in metastatic colorectal cancer. J Immunother Cancer. (2021) 9:1–14. doi: 10.1136/jitc-2021-002844 PMC8317124 34315821

[8] Bortolomeazzi M Keddar MR Montorsi L Acha-Sagredo A Benedetti L Temelkovski D. Immunogenomics of colorectal cancer response to checkpoint blockade: analysis of the KEYNOTE 177 trial and validation cohorts. Gastroenterology. (2021) 161:1179–93. doi: 10.1053/j.gastro.2021.06.064 PMC8527923 34197832

[9] Lei JX Wang R Hu C Lou X Lv MY Li C. Deciphering tertiary lymphoid structure heterogeneity reveals prognostic signature and therapeutic potentials for colorectal cancer: a multicenter retrospective cohort study. Int J Surg. (2024) 110:5627–40. doi: 10.1097/JS9.0000000000001684 PMC11392219 38833363

[10] Nicolini A Ferrari P. Involvement of tumor immune microenvironment metabolic reprogramming in colorectal cancer progression, immune escape, and response to immunotherapy. Front Immunol. (2024) 15:1353787. doi: 10.3389/fimmu.2024.1353787 39119332 PMC11306065

[11] Hong Y Chen Q Wang Z Zhang Y Li B Guo H. Targeting nuclear receptor coactivator SRC-1 prevents colorectal cancer immune escape by reducing transcription and protein stability of PD-L1. Adv Sci (Weinh). (2024) 11:e2310037. doi: 10.1002/advs.202310037 38953362 PMC11434141

[12] Li JJ Wang JH Tian T Liu J Zheng YQ Mo HY. The liver microenvironment orchestrates FGL1-mediated immune escape and progression of metastatic colorectal cancer. Nat Commun. (2023) 14:6690. doi: 10.1038/s41467-023-42332-0 37872170 PMC10593839

[13] Westcott PMK Sacks NJ Schenkel JM Ely ZA Smith O Hauck H. Low neoantigen expression and poor T-cell priming underlie early immune escape in colorectal cancer. Nat Cancer. (2021) 2:1071–85. doi: 10.1038/s43018-021-00247-z PMC8562866 34738089

[14] Zhou X Wang G Tian C Du L Prochownik EV Li Y. Inhibition of DUSP18 impairs cholesterol biosynthesis and promotes anti-tumor immunity in colorectal cancer. Nat Commun. (2024) 15:5851. doi: 10.1038/s41467-024-50138-x 38992029 PMC11239938

[15] Aria M. bibliometrix: An R-tool for comprehensive science mapping analysis. J Informetrics. (2017) 11:959–75. doi: 10.1016/j.joi.2017.08.007

[16] Chen C. Searching for intellectual turning points: progressive knowledge domain visualization. Proc Natl Acad Sci U S A. (2004) 101 Suppl 1:5303–10. doi: 10.1073/pnas.0307513100 PMC387312 14724295

[17] van Eck NJ Waltman L. Software survey: VOSviewer, a computer program for bibliometric mapping. Scientometrics. (2010) 84:523–38. doi: 10.1007/s11192-009-0146-3 PMC2883932 20585380

[18] Cervantes-Villagrana RD Albores-García D Cervantes-Villagrana AR García-Acevez SJ. Tumor-induced neurogenesis and immune evasion as targets of innovative anti-cancer therapies. Signal Transduct Target Ther. (2020) 5:99. doi: 10.1038/s41392-020-0205-z 32555170 PMC7303203

[19] Jiang J Liu F Cui D Xu C Chi J Yan T. Novel molecular mechanisms of immune evasion in hepatocellular carcinoma: NSUN2-mediated increase of SOAT2 RNA methylation. Cancer Commun (Lond). (2025). doi: 10.1002/cac2.70023 PMC12328098 40227950

[20] Lakhani NJ Chow LQM Gainor JF LoRusso P Lee KW Chung HC. Evorpacept alone and in combination with pembrolizumab or trastuzumab in patients with advanced solid tumours (ASPEN-01): a first-in-human, open-label, multicentre, phase 1 dose-escalation and dose-expansion study. Lancet Oncol. (2021) 22:1740–51. doi: 10.1016/S1470-2045(21)00584-2 34793719

[21] Li J Duran MA Dhanota N Chatila WK Bettigole SE Kwon J. Metastasis and immune evasion from extracellular cGAMP hydrolysis. Cancer Discov. (2021) 11:1212–27. doi: 10.1158/2159-8290.CD-20-0387 PMC8102348 33372007

[22] Chen X Ma Z Yi Z Wu E Shang Z Tuo B. The effects of metabolism on the immune microenvironment in colorectal cancer. Cell Death Discov. (2024) 10:118. doi: 10.1038/s41420-024-01865-z 38453888 PMC10920911

[23] Liu C Yao Z Wang J Zhang W Yang Y Zhang Y. Macrophage-derived CCL5 facilitates immune escape of colorectal cancer cells via the p65/STAT3-CSN5-PD-L1 pathway. Cell Death Differ. (2020) 27:1765–81. doi: 10.1038/s41418-019-0460-0 PMC7244707 31802034

[24] Zhu J Zhang J Lou Y Zheng Y Zheng X Cen W. Developing a machine learning-based prognosis and immunotherapeutic response signature in colorectal cancer: insights from ferroptosis, fatty acid dynamics, and the tumor microenvironment. Front Immunol. (2024) 15:1416443. doi: 10.3389/fimmu.2024.1416443 39076986 PMC11284049

[25] Li S Yan G Yue M Wang L. Extracellular vesicles-derived microRNA-222 promotes immune escape via interacting with ATF3 to regulate AKT1 transcription in colorectal cancer. BMC Cancer. (2021) 21:349. doi: 10.1186/s12885-021-08063-5 33794833 PMC8017736

[26] Clancy JW D’Souza-Schorey C. Tumor-derived extracellular vesicles: multifunctional entities in the tumor microenvironment. Annu Rev Pathol. (2023) 18:205–29. doi: 10.1146/annurev-pathmechdis-031521-022116 PMC10410237 36202098

[27] Xiao H Du X Tao Z Jing N Bao S Gao WQ. Taurine inhibits ferroptosis mediated by the crosstalk between tumor cells and tumor-associated macrophages in prostate cancer. Adv Sci (Weinh). (2024) 11:e2303894. doi: 10.1002/advs.202303894 38031260 PMC10797466

[28] Anderson P Aptsiauri N Ruiz-Cabello F Garrido F. HLA class I loss in colorectal cancer: implications for immune escape and immunotherapy. Cell Mol Immunol. (2021) 18:556–65. doi: 10.1038/s41423-021-00634-7 PMC8027055 33473191

[29] Kobayashi Y Niida A Nagayama S Saeki K Haeno H Takahashi KK. Subclonal accumulation of immune escape mechanisms in microsatellite instability-high colorectal cancers. Br J Cancer. (2023) 129:1105–18. doi: 10.1038/s41416-023-02395-8 PMC10539316 37596408

[30] Na HY Park Y Nam SK Lee KS Oh HK Kim DW. Expression of human leukocyte antigen class I and β2-microglobulin in colorectal cancer and its prognostic impact. Cancer Sci. (2021) 112:91–100. doi: 10.1111/cas.v112.1 33159376 PMC7780028

[31] Chang Y Chang M Bao X Dong C. Advancements in adoptive CAR immune cell immunotherapy synergistically combined with multimodal approaches for tumor treatment. Bioact Mater. (2024) 42:379–403. doi: 10.1016/j.bioactmat.2024.08.046 39308543 PMC11415837

[32] Wu J Shi K Chao W Qin Z Hu Y Yang Y. Artificially modified NK cell-based synergistic immuno-gene-photodynamic therapy for cancer. Int J Nanomed. (2024) 19:12323–42. doi: 10.2147/IJN.S481368 PMC11587796 39588262

[33] Tzatzarakis E Hissa B Reissfelder C Schölch S. The overall potential of CD47 in cancer immunotherapy: with a focus on gastrointestinal tumors. Expert Rev Anticancer Ther. (2019) 19:993–9. doi: 10.1080/14737140.2019.1689820 31686549

[34] Wang X Luo X Chen C Tang Y Li L Mo B. The Ap-2α/Elk-1 axis regulates Sirpα-dependent tumor phagocytosis by tumor-associated macrophages in colorectal cancer. Signal Transduct Target Ther. (2020) 5:35. doi: 10.1038/s41392-020-0124-z 32296015 PMC7156469

[35] Hsieh RC Krishnan S Wu RC Boda AR Liu A Winkler M. ATR-mediated CD47 and PD-L1 up-regulation restricts radiotherapy-induced immune priming and abscopal responses in colorectal cancer. Sci Immunol. (2022) 7:eabl9330. doi: 10.1126/sciimmunol.abl9330 35687697 PMC9373855

[36] Xu L Che S Chen H Liu Q Shi J Jin J. PPARγ agonist inhibits c-Myc-mediated colorectal cancer tumor immune escape. J Cell Biochem. (2023) 124:1145–54. doi: 10.1002/jcb.v124.8 37393598

[37] Li Y Shen Z Chai Z Zhan Y Zhang Y Liu Z. Targeting MS4A4A on tumour-associated macrophages restores CD8+ T-cell-mediated antitumour immunity. Gut. (2023) 72:2307–20. doi: 10.1136/gutjnl-2022-329147 PMC10715532 37507218

